# Heart failure outpatient clinics resources in Italy: a viewpoint of Italian Society of Cardiology organization

**DOI:** 10.1007/s10741-024-10480-0

**Published:** 2025-01-08

**Authors:** Alberto Palazzuoli, Piergiuseppe Agostoni, Savina Nodari, Stefania Paolillo, Pasquale Perrone Filardi

**Affiliations:** 1 Cardiovascular Diseases Unit, Cardio Thoracic and Vascular Department, Le Scotte Hospital, University of Siena, Viale Bracci 53100, Siena, Italy; 2 Centro Cardiologico Monzino, IRCCS, Milan, Italy; 3 Department of Clinical Sciences and Community Health, Cardiovascular Section, University of Milan, 20122 Milan, Italy; 4 Department of Medical and Surgical Specialities, Radiological Sciences and Public Health, University of Brescia Medical School, Brescia, Italy; 5 Cardiology Unit, Department of Advanced Biomedical Sciences, University Federico II Naples, Naples, Italy

**Keywords:** Heart failure clinics, Management, Health resources, Organization models

## Abstract

The current paper reports the model organization, level of health care, and type of medical and research activities related to the existing heart failure centers of the Italian Society of Cardiology. Of note, we conduced an internal survey among the members of heart failure working group and related hospital and territorial sites about the quality of care and assistance levels according to the local hospital resources and type of diagnostic therapeutic and management resources. Thirty-two hospital ambulatorial structures have been identified, the centers were equally distributed within the national ground, with similar concentration between north and south regions of the Italian country. We distinguished three different levels of organization: (1) basal territorial clinics in which patients with suspected or already diagnosed heart failure (HF) are initially identified and screened; (2) intermediate clinics in which HF patients can be routinary followed by HF specialists supported by a dedicated staff including imaging and arrythmologist experts, and interventional cardiologist; (3) advanced clinics composed by all the technical and staff resources capable of guarantying repetitive invasive assessment, continuous invasive monitoring, dedicated telemedicine structures focused on more advanced HF management integrated by heart transplantation or mechanical assistance programs. Different type of assistance is supported by a relevant number of research activity primarily conducted by the Italian Society of Cardiology or spontaneous studies arranged by HF specialist members. The number of HF centers has increased over the past few decades in proportion to the progressive rise in HF diagnoses and associated hospitalization. The expansion of ambulatory structures has been facilitated by an increasing socioeconomic and research influence. The quality of HF services in Italy could be raised by improving the network and connections between HF specialists, general practitioners (GPs), caregivers, and other specialists frequently working in this field.

## Introduction

Heart failure (HF) is the leading cause of mortality among the whole spectrum of cardiovascular (CV) diseases with a high rate of recurrence leading to a high rate of hospitalizations after the first diagnosis [1, 2]. Nevertheless, many differences have been observed according to different countries, regions, and models of care management. Additionally, the prognosis may vary according to NYHA class, perfusion and congestion status, HF aetiology, baseline general condition, HF aetiology, treatment adherence, and response [3–5]. Therefore, outcome and hospitalization are highly variable depending on the primary cause of destabilization (arrhythmic storm, pump failure, or systemic events). Prediction of recurrence modality and reasons of deterioration may facilitate decisions about specific medications or devices. This framework leads to high health system costs and social commitment: The last Italian survey conduced by the Institute of Health found that around 2% of the whole population experienced this syndrome with around 200,000 hospital admission per year accounting the second cause of hospitalization after delivery. It is the first cause of mortality among all CV diseases with mortality rate after 1 month since hospitalization of 10% [6, 7]. The HF is much more prevalent in elderly people, frailty patients with high comorbidity burden. A recent report based on administrative data, collected from 2003 to 2018, showed that in Lombardia, a region with almost 10 million inhabitants, while the age and complexity of patients increased in those admitted for acute HF more recently, in-hospital and 30-day mortality risks were similar over time (relative risk for trend 1.00 [95% CI 0.99–1.01] and 1.00 [95% CI 0.98–1.01], respectively), while that of 30-day re-hospitalization decreased progressively (hazard ratio for trend 0.86 [95% CI 0.84–0.88]) [8]. Around half of hospitalization for HF is due to systemic or extracardiac reasons such as infections, pneumonia, chronic lung disease recurrency, anemia, metabolic causes, and renal function deterioration [5, 9]. Current features make HF as a disease with arduous prognostic prediction and difficult to intercept during the follow-up. Because of its multifaceted aspects and different phenotypes with various associated diseases, there is a need for HF center distribution across all the geographical sites of the country and diffuse outpatient clinic capillarization in order to reach a good network and optimal management according to HF typology severity and patient status [10]. In this framework, we have organized a national survey contacting the associated SIC center to investigate the local structure organization, level of management, connection with hub hospitals, and type of clinical management. In this analysis, we did not study specific patient aspect such as HF typology severity and demographic characteristics.

## Aims and statute of Italian Society of Cardiology

The Italian Society of Cardiology (SIC) is an academic association born to facilitate diffusion and awareness of the main CV diseases with the principal aim to promote scientific meeting, publication, and scientific updating among affiliates and members. Further scope of the Society is to increase scientific education learning and debates by the production of documents, scientific reports, and position papers dedicated to CV diseases [11]. The Society is composed of several research working groups that deal with specific topics according with the proper attitude, nomenclature, and scope. In this regard, the heart failure working group of Italian Society of Cardiology (SIC) facilitates the diffusion and knowledge of recent research and guidelines in the HF field, and it promotes proper scientific activity and scientific debate congresses to improve a national network and physician relationship. Cardiologic education and fellow training activity and collaborative facilitation between members and other societies potentially involved in HF are among the scopes of SIC. Additional scopes are: to perform initiatives about health and social organization involving patients, caregivers and health personnel, facilitating the cultural spread and knowledge of HF and potential management strategies. Other initiatives endorsed by the SIC account the collaboration with government regional institutions, Italian Drug Agency (AIFA), international scientific societies to arrange formative programs for epidemiological analysis, and health organization models. Current activities are supported by heart failure specialists working into the national territory where the health assistance is guaranteed by various outpatient clinic distribution and skills. (Table 1).

**Table 1:** Main scopes and activities of the Italian Society of Cardiology (SIC) heart failure study group

Aims of Italian Society of Cardiology- Heart Failure Study Group
• Promote awareness of main cardiovascular diseases with specific interest for heart failure investigation
• Improve scientific education learning and debate in heart failure topic
• Produce scientific documents, position papers, and reports addressed to the last innovations in heart failure
• Spread scientific guidelines and knowledge in heart failure
• Facilitate scientific data diffusion center collaboration and national network
• Improve cardiology education and fellow training activity by meetings lessons and tutorial initiatives
• Promote the cultural spread and knowledge of HF among patients caregivers and social organizations
• Collaborate with regional national and government Institutes to promote optimal management, prevention, and organization models

## Outpatient clinics organization and diagnostic resources

During the last decades, there has been special interest in healthcare delivery methods that aim to improve HF control. Whereas, the significant limitations come from substantial etherogeneithy regarding the resources of the HF clinics, the related services, and interventions provided. Our objective was to ascertain significant information gaps by conducting a field evaluation in which we evaluated actual care for HF patients in outpatient clinics across the Italian territory. Our particular objectives were to (1) investigate the clinical effectiveness and resources of a cohort of HF patients treated at specialized HF clinics according to location and (2) determine which aspects of the service models equipped by HF clinics were linked to the intervention offered. Accordingly, HF clinics were defined as one that contained at least one doctor and a nurse, one of whom had specific training and interest in HF; interviews were carried out at the HF clinic after they were identified.

**Fig. 1:**
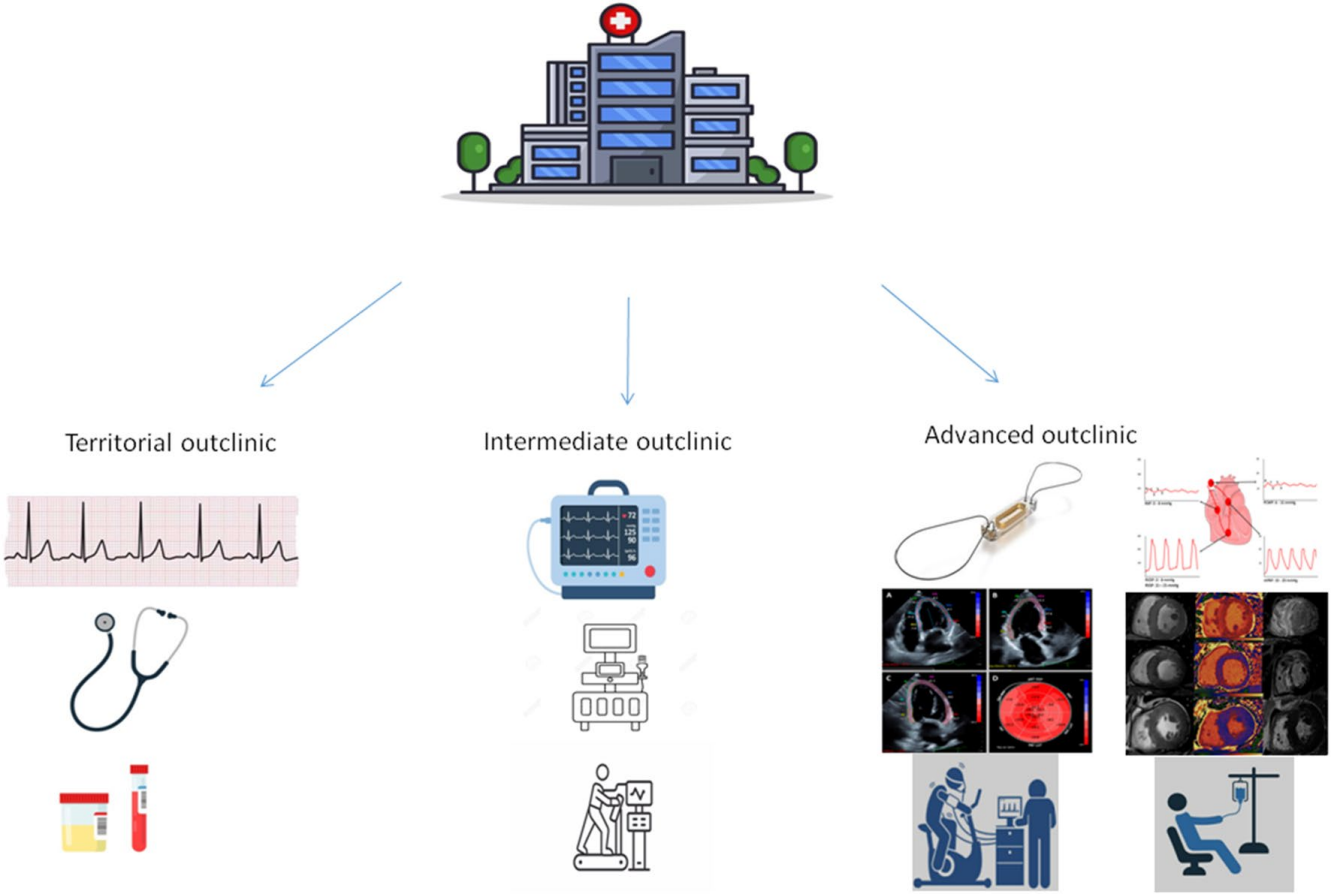
Three levels of outpatient clinics related to the country site, health resources, hospital technology, and type of intervention

Outpatient clinics are prevalently inserted inside hospital structures to facilitate early evaluation after discharge and follow-up management. There are some clinics located outside the hospital in a peripheral territory far away from tertiary centers which serves as spoke sites for initial HF evaluation. SIC HF outpatient clinics are 32 and they are composed by mixed medical staff (academic and hospital personnel) integrated with fellows and dedicated nurse staff. All sites are uniformly distributed across Italian country with a good balance among the north center and south regions; however, the most relevant clinics are located in the north of Italy. According to the level of care and patients typology, we can distinguish among three different structure levels:

- Territorial ambulatory in which patients with suspected HF diagnosis are evaluated by clinical assessment ECG, initial ultrasound evaluation, and laboratory examination including natriuretic peptide assay, emocrome electrolyte renal and liver function, glycemic and lipidic profile.
- Intermediate structure dedicated to a more specific evaluation in patients with known HF accounting the above-cited resources dedicated staff including echocardiographic and arrythmologist experts, noninvasive hemodynamic monitoring tools, holter ECG and blood pressure instruments, functional patients evaluation with exercise or pharmacological tests ( six min walking test, exertional ECG, exertional echo). Current clinics are commonly associated with nuclear medicine and radiologic wards for integrative imaging examination. These types of instruments and tools are still available for rehabilitation centers dedicated to post-discharged subjects, patients submitted to cardiac surgery, and those with prolonged bed stay and reduced mobility.
- Advanced structures composed by all the technical and staff resources integrated by hemodynamic wards for right cardiac catheterization capable of guarantying repetitive invasive assessment, continuous invasive monitoring (cardio-mems), dedicated telemedicine structure. Cardio-pulmonary test, spirometric, and gas exchange evaluation is an additional tool for more precise patient screening and risk assessment. A more complete imaging staff composed of dedicated radiologists and cardiologists for cardiac CT and RM execution is also available. However, in Italy, because of a law decree, the management of these diagnostic exams needs a radiology supervision and approval [12]. These clinics are more strictly associated with in-hospital wards and dedicate routes for HF patients in order to facilitate hospital admission, daily infusional treatment, and recurrent management (Fig. 1).

The former clinics are managed and attended by both cardiologist and internal medicine doctors having a specific skill on HF management, whereas the latter structures dedicated to more severe and advanced HF degree, operate by a direct cardiologist and intensive cardiologist work. Different types of assistance are supported by a relevant number of cases studied and research activity by the Italian heart failure allowed a strong presence of the Italian heart failure scientist in several studied including, MECKI score data, OPTIPHARM- HF, and ECLIPSE HF registries [13–16].

## Type of patients followed in SIC clinics

The level of assistance and health care resources changes according to patient status, HF typology, HF aetiology, age, comorbidities burden, and disease journey. Indeed younger patients with a recent history of myocardial infarction or dilatative cardiomyopathy (DCM) and specific familiar heritage are usually treated and followed in specific centers independently from their clinical status, NYHA class, arrhythmias occurrence, or therapy response. These patients are healed for eventual device therapy (ICD, CRT) application, not only in secondary prevention but also in primary prevention if judged at high risk for HF recurrence or high arrhythmic burden. Otherwise, older patients(> 75 years) with elevated frailty level, associated systemic diseases, long HF history, and advanced NYHA class, even with severe systolic dysfunction and low ejection fraction (EF), are generally managed from the intermediate center applying a conservative strategy addressed to drug optimization and repetitive surveillance. Among these two conditions, there are several “gray zone” subjects indifferently followed by all three mentioned outpatient structures depending on local health resource, geographic location, caregiver disposal, hospital distance, patients life-self, and choice [17]. In theory, each clinic should take charge of subjects in relation to HF stages, clinical history, worsening episodes, and HF phenotypes. Based on these rules, territorial ambulatory may be mostly dedicated to HF prevention with specific attention on CV risk factors controls, lifestyle habits correction, and general patient condition. Subjects in stages A and B of AHA classification, patients with evidence of initial cardiac structural and functional sisto-diastolic dysfunction, and those with moderate NP level increase without evidence of deterioration may be managed in these structures.

The intermediate structure should be dedicated to patients with HF evidence, previous episodes of HF hospitalization, those with frank systolic dysfunction (ejection fraction EF < 40%) or evidence of advanced diastolic dysfunction reflecting HFpEF diagnostic criteria, patients with reduced physical activity in NYHA class > I and those with baseline NP (BNP > 100 and NTproBNP > 400 pg/ml, respectively). Additionally, subjects with intermediate or questioned arrhythmic risk, those with left bundle branch block (LBBB), patients, and those needing therapy titration or diuretic dose adjustment should be observed by the current structure [18, 19]. These clinics may serve for early assessment after hospitalization within 4 weeks from discharge in subjects who are not candidates for electrical or mechanical devices [20]. Finally, patients in which all attempts of treatment have been performed with persisting symptoms, cachectic condition, severe reduction of physical activity, poor quality of life, and those who do not tolerate traditional treatment who can be classified for palliative care, are in charge to intermediate clinic with the integration of other specialists such as a psychologist, geriatrician, nutritionist, or physiotherapist according to the underlying conditions [20, 21].

Advanced clinic is usually dedicated for patients with more severe HF forms, advanced NYHA class patients candidate for heart transplantation (HTx) and left ventricle assistance device (LVAD), patients previously submitted to CRT or ICD implantation, those needing continuous invasive or noninvasive monitoring prone to hemodynamic status deterioration with reduced cardiac output, persistent LV filling pressure and wedge pressure increase, and mean pulmonary and central venous pressure persistently elevated [22]. Subjects with recurrent episodes of pulmonary and systemic congestion experiencing diuretic resistance, needing elevated loop diuretic dose or diuretic combination awaiting for invasive treatment represent a typical phenotype treated in this ward [23]. Subjects with evidence of ventricular repetitive tachycardia leading to hemodynamic impairment, people with planned ICD introduction, and those suitable for ventricular ablation, ventricular pacing, or electric stimulation may be managed in these structures. Finally, subjects needing periodical treatment (weekly monthly) with infusional diuretic, inotropes, lusitropes, and renal replacement treatment (RRT) such as plasmapheresis or ultrafiltration are candidates for these structures [24]. These types of clinics are usually connected with HTx centers or specific HF-dedicated cardiology wards, and they work as hub reference sites for less advanced structures [23, 25].

## Type of treatment endorsed by the Italian government

Patients affected by HF can benefit from all agents endorsed by guidelines independently from HF phenotype severity and onset mechanism. The list of drugs includes betablocker, renin angiotensin aldosterone system inhibitor (RAASi) including both angiotensin conversing enzyme inhibitors ( ACE-i) or angiotensin receptor 1 inhibitors (AT-1i), mineral corticoid antagonist (MRA), and sodium glucose co-transporter- 2 inhibitor (SGLT2). Nevertheless, some aspects and differences with respect to North American and other European countries need to be highlighted: the use of sacubitril /valsartan (S/V) is endorsed only for patients who experienced HFrEF and HFmEF but not in those with EF > 50%. Additionally, the SGLT2i admitted for HF treatment are only Dapaglifozin and Empaglifozin in subjects with glomerular filtration rate > 30 ml/min/m2 regardless of EF values and diabetes occurrence. HF specialist cannot prescribe antidiabetic combination therapy, glucagon-like peptide 1 antagonist (GLP1a), or dipeptidyl peptidase-4 enzyme inhibitor (DPP-4i) that can be currently administered only by diabetologists in subjects with diabetes and evidences or high CV risk. A recent ministerial note admitted the cardiology prescription for sitagliptin in subjects with severe metabolic alterations, evidence of cardiac dysfunction, and /or diabetes. Importantly, all the new drugs ( S/V, SGLT2i, and GLP-1) are under the surveillance of the Italian Drug Agency (AIFA) and they need a specific document including patient data, reasons for prescription, renal function, and electrolyte measurement executed within 3 months from data prescription. Similarly, most recent potassium binders (patiromer and sodium zirconium cyclosilicate) are admitted for HF treatment only in patients with hyperkalemia and associated chronic kidney disease (CKD) II and III stage taking RAASi treatment. The more common diuretic therapy such as loop diuretics (furosemide and torasemide), acetazolamide, metolazone, and thiazides can be administered in patients with congestion evidence with the exception of bumetanide that is not diffused in Italian country. Finally, vericiguat prescription is recently introduced in patients with recurrent hospitalization episodes and worsening HF with reduced EF significant increase of NP levels only in addition to the on-top treatment as second-line therapy.

## The role of telemedicine

With recent advances in telecommunication technologies, HF patients may benefit from new management alternatives to the traditional medical assessment. Internet communication technology (ICT) has enormous promise for helping patients affected by chronic disease. However, there are different viewpoints about the benefits and effectiveness of ICT interventions for older persons living with chronic conditions. Two meta-analyses suggest that telemedicine can reduce morbidity and hospitalization in such patients [26, 27]. Conversely, the results of TIM-HF suggest that telemedicine applied to stable, optimally treated, ambulatory chronic HF patients, no significant advantages in adverse events was found [28]. Current digital tools and applications may be appropriately used to manage HF, despite some issues that require close consideration and further analyses: the accuracy and safety of these telemonitoring tools in HF diagnosing, the effectiveness in HF management compared to traditional face-to-face doctor–patient interaction, the sensitivity of measured parameters to recognize effective hemodynamic deterioration. In addition, the telemedicine efficacy could depend on patient profile, knowledge of the internet and confidence with wireless systems, the adequacy of application used, and the quality of measured variables [29, 30].

Notably, telemedicine approaches range from computer-based support systems measuring noninvasive variables to advanced programs led by nurses and physicians assessing pulmonary pressure, right atrial pressure, and fluid status by specific devices. The profile of patients who can potentially benefit from telemedicine remains unclear: Standardization and appropriate classification of telemedical systems should be investigated in adequately powered randomized clinical trials [31]. Individual characteristics of patients with HF obtained from the analysis of a large number of various HF populations may allow the identification of those patients at higher risk of negative outcomes who could most likely benefit from individualized medical treatments [32]. Because of these weaknesses and relevant costs to build appropriate telemedicine networks that enable to provide a significant HF events reduction, the role of telemedicine in Italy remains doubtful and not extensively applied. Most of the centers adopted a single system built based on local exigences and administrator skills, evaluating traditional parameters such as blood pressure heart rate, urinary output, weight change, and ECG trace derived by 1 or 2 leads associated with a self-quality appraisal evaluated by simple questionnaire connected by simple system or by phone. Current data are periodically transmitted and registered by dedicated nurse staff. However this application is available for a minor percentage of HF population, those judged at intermediate-high risk, experiencing WHF, and younger population < 70 years old. Whereas in more advanced HF patients, monitoring is usually guaranteed by an intracardiac device placed during hospital permanence capable of identifying both arrhythmic and pump failure events. Similarly, chronic HF patients or de novo patients with severe systolic function reduction, those screened for LVAD or Htx, may benefit from cardio-mems or similar devices to early ascertain eventual congestion occurrence and to monitor intracardiac pressures [33, 34]. In this setting, data are periodically transmitted (every week) to adequate the optimal treatment and diuretic dose changes. Overall, the percentage of patients followed with telemedicine in Italy remains inappropriate compared to the other industrialized countries and the whole HF population (at least 10%). The majority of contacts and information between patients and clinics in the more peripheral centers remains linked to direct visit or phone call [35]. Beyond remote invasive and traditional monitoring, several alternative methods providing health status information may be easily obtained by mobile health-dedicated apps offering relevant opportunities for improving self-care behaviors [36]. Communication with clinics may occur by three different systems look into three benefits in terms of self-care behaviors: (1) Internet use to search for HF information; (2) familiarity with mobile health apps and gadgets; and (3) perceptions of utilizing activity trackers or smartwatches to aid in heart failure self-care. Despite potential advantages and facilities, several gaps still exist about the diffusion of current screening: identified age-related hurdles to implementing ICT therapies, such as cognition, motivation, physical limitations, and perception, which hindered the use of ICT interventions among older persons with chronic conditions. Additive barriers are patients’ trust in their physicians, lack of necessity for mobile health devices, financial hurdles to activity tracker and wristwatch ownership, diffidence in tracking and remainder benefits, and doubt about their potential owing to lack of information. Hence, clinicians and health care providers may be more aware to convince patients to use ICT interventions in addition to routine clinic visits [37]. More sustainable methods of financing and reimbursement will also be necessary for the widespread use of ICT applications. Finally, the right use of ICT may include common learning courses involving both clinicians and caregivers into standard medical care.

## Future perspective and organization models

Current HF outpatient clinics encompass a wide range of patients and disease severity according to single HF clinic resources, connection with neighboring centers, regional and local health legislative rules, and caregiver organization. Over the last decades, there was a relevant increase in center numbers with regard to the raised number of HF diagnosis and related hospitalization costs in Italian country. A growing socio-economic and research impact contributed to the extension of ambulatorial structures and enhanced the interest of both politicians and physicians. Anyway, the objectives of the health system and doctors involved in this setting do not perfectly coincide: the administrator’s aim is substantially focused to reduce disease-related costs and expenses including pharmaceutical, social, medical, and environmental aspects. Otherwise, research and physician activity is focused on patients’ quality of life improvement, life expectancy prolongation, and treatment optimization using all technical and human instruments potentially available. In other words, we observe on one side a persistent requirement to reduce public outgoings and to concentrate the financial resources towards the younger patients with more chances of disease recover; on the other side, a continuous increase of HF events occurring in more elderly patients, frailty population requiring medical nurses and social assistance, would need a more financial disposal from government. In this framework, an expansion of in-patient and outpatient HF clinics should be desirable with a more structured system focused on the prevention and treatment of disease exacerbations and the promotion of patient self-empowerment as main objectives [35]. Notably, some models and future investigations may be arranged. A national survey investigating the precise level of care, quality of diagnostic instruments, patient profiles, center availability, services provided, and follow-up modalities for each SIC outpatient clinics may enhance the standard of care and homogenize management strategy. Additionally, territorial staff assistance for patients unable to reach hospital centers should be of worth. Similarly, a more diffuse day hospital service for patients needing intravenous infusions and those in palliative care could significantly decrease hospitalization and related costs and ameliorate patients’ perception of illness [38]. These features could be improved by an effective remote monitoring application, and through personalized medical algorithms based on previous patients’ risk stratification, optimal visit planning, disease severity, and specific management strategy. A better network and connection among HF specialists, general practice (GP) doctors, caregivers, and other specialists often involved in this field, could improve the quality of HF services. This type of organization should be possible at a low cost by employing fellows and younger doctors in GP ambulatories or by their direct involvement in patients’ home monitoring, assessment, and wireless data acquisition (Table 2). The construction of registries and trials analyzing the characteristics of Italian HF population, diagnostic tools employed, therapeutic choices, and follow-up data may bring to a more accurate recognition of the demographic, clinical, and therapeutic characteristics of Italian patients affected by HF [39–41]. Extensive applications of these strategies could drive towards better resource utilization and more appropriate outpatient clinical service.

**Table 2:** Strengths and limitations of the current Italian population affected by heart failure can guarantee a good quality of treatment despite some gaps about monitoring and check-up

Strength	Weakness
Increased number of HF centers in national country	Poor awareness among population particularly in peripheral sites
Diffuse capillarization and spread of HF clinics with better monitorization compared with previous decades	Reduced financial resources for advertising media social and medical campaign
Quality of life and life expectancy improvement with improved prevention for recurrences episodes and tailored therapy	Invasive and noninvasive monitoring limited for younger patients excluding other advanced or high-risk subjects
Optimized treatment endorsed by international guidelines warranted, specific HF high quality center for each region	Delay in outpatient visit planning and collection due to different regional health system organization
Good network for patients with advanced HF and homogeneous selection for those deserving Heart transplantation or device implantation	Reduced resources for home visit, nurses, and social assistance for elderly patients with reduced mobilization capacity and poor familiar support
Registries implementation to assess the prevalence of HF subtypes, management, and timing course	Poor resource for telemedicine spread and system modality uniformities
Organization of outpatient center for daily treatment and monitorization in advanced heart failure	Limited organization for palliative care and peripheral assistance needing optional health personal distribution
